# Transient midventricular ballooning: a case report and review of the literature

**DOI:** 10.1186/1757-1626-1-431

**Published:** 2008-12-30

**Authors:** Jamil Y Abuzetun, Senthil K Thambidorai, Ribhi Hazin, Manar Suker, Claire B Hunter

**Affiliations:** 1 Creighton University Medical Center, Division of Internal Medicine, 601 N. 30th Street Suite 5850 Omaha, NE 68131, USA; 2 Creighton University Cardiac Center, 3006 Webster Street, Omaha, NE 68131, USA; 3 Harvard University's Kennedy School of Government & Policy, 79 JFK St Cambridge, MA 02138, USA

## Abstract

**Background:**

We describe a case of transient left midventricular ballooning in 68-year-old male patient presented with picture of acute coronary syndrome.

**Case presentation:**

The left ventriculogram showed mid ventricular akinesis and dilatation along with hypercontractile apex and basal segments. Follow up echocardiogram after one month showed resolution of wall motions abnormalities and normalization of the left ventricular function.

**Conclusion:**

This is considered as a new variant of previously reported transient left ventricular apical ballooning; the only difference in our case is the location of wall motions abnormalities.

## Case presentation

**Figure 1:**
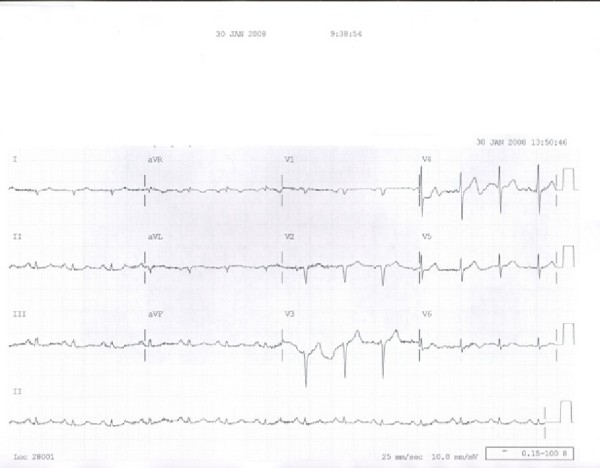
**EKG demonstrating Q waves in lateral leads (I and AVL) and poor R wave progression in the precordial leads**.

**Figure 2:**
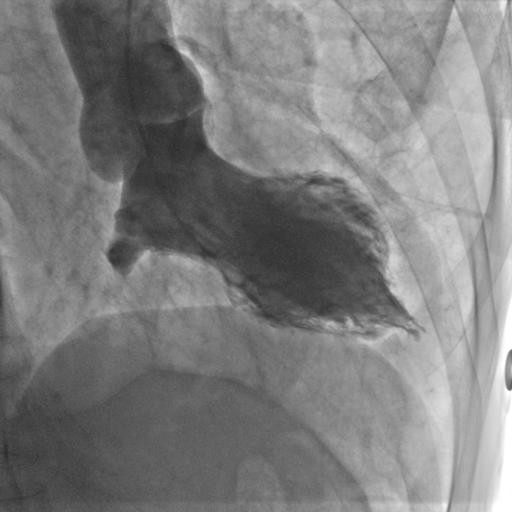
**Left ventriculogram demonstrating midventricular akinesis with hypercontractile apex and basal segments**.

A 68-year-old black male with past medical history significant for chronic obstructive pulmonary disease, hypothyroidism, and tobacco abuse presented to the emergency department with acute onset of retrosternal chest pain. The pain was described as 10/10 in severity with no radiation, associated with shortness of breath, sweating and palpitation. His home medications included albuterol, formoterol and synthroid. The patient complained of being under a great deal of stress since recently losing his job. Physical examination revealed a well nourished 68-year old male with a blood pressure of 97/62 mmHg, heart rate of 72/min, temperature 97.6F, and respiratory rate of 18/min. Heart exam revealed normal heart sounds without murmurs, gallop or rub. His lungs were clear to auscultation. The rest of the physical examination was unremarkable. Electrocardiogram showed Q waves in lateral leads (I and AVL) and poor R wave progression in the precordial leads [Fig 1]. Laboratory work up showed borderline cardiac enzymes with peak troponin 0.41. He was treated with aspirin, low molecular weight heparin, metoprolol, and nitroglycerin. The pain was alleviated with the therapy and a transthoracic echocardiogram revealed ejection fraction 30 to 35%. Subsequently he underwent left heart catheterization which showed normal coronary arteries, mid ventricular akinesis with hypercontractile apex and basal segments [Fig 2]. Patient was chest pain free through the course of hospitalization. Follow up echocardiogram after one month revealed normal left ventricular function and resolution of the wall motion abnormalities.

## Discussion

Transient left ventricular apical ballooning or Tako-tsubo cardiomyopthy first described in Japan in 1991. Subsequently multiple cases have been reported in the western literature. The incidence is around 1% in patients presented with acute coronary syndrome and positive troponin [1]. It is much more common in postmenopausal female though it can happen in males and premenopausal female [2]. Patients with transient left ventricular dysfunction usually present with acute onset of chest pain at rest, shortness of breath along with ST segment elevation in the majority of the cases, non-specific ST-T wave changes, pathological Q wave have been described. Mild increase in the cardiac enzymes is usually the case and all these features make it indistinguishable form acute myocardial infarction and warrant more investigation to make the diagnosis. Further more transient left ventricular apical ballooning is characterized by normal epicardial arteries or non obstructive disease along with wall motions abnormalities involving the mid and the apical segments sparing the basal segment [2,3].

When this clinical syndrome was first described it was refer to as transient left ventricular apical ballooning since all the patients had characteristic akinesia and ballooning of the apex, midventricular dysfunction and a normal or hypercontractile basal segment as evident by the left venticulogram. Hurst et al [4], reported a new variant of this clinical syndrome in four female patients who presented with symptoms suggestive of acute coronary syndrome. Left heat catheterization revealed normal epicardial arteries, while ventriculogram showed midventricular akinesis and dilatation with hepercontractile apex and base similar to the patient we presented above. This variant is similar in term of clinical presentation and outcome to the transient left ventricular apical ballooning. In contrast the apex is hypercontractile and the midventricular segments demonstrate ballooning and akinesia.

The underlying mechanism of transient left ventricular dysfunction still poorly understood. One of the postulated mechanisms is microvascular dysfunction which has been identified in two third of the patients presented with apical ballooning syndrome, impaired myocardial perfusion despite normal epicardial flow has been documented based on coronary angiography[5]. Catecholamine levels ware found to be high in patients with left ventricular apical ballooning compared to patients with acute myocardial infarction. The levels remain elevated even after one week after the initial presentation. The postulated mechanisms of catecholamine induced cadiomyopathy include mutivessel epicardial spasm, micovascular dysfunction or direct myocytes injury [6].

Ideal treatment of transient left ventricular ballooning dysfunction is not well studied. Patients receive the standard therapy for acute coronary syndrome at presentation till this syndrome is recognized at the time of coronary angiography. Logically beta-blocker can be considered the cornerstone of treatment if no contraindications exist. Heart failure is treated with standard medical therapy including diuretics. The role of aspirin is debatable in the absence of atherosclerosis. The syndrome can be complicated with left ventricular apical mural thrombosis and require appropriate therapeutic anticoagulation. Prevention of mural thrombus formation should be seriously considered if left ventricular function is severely depressed. Formation of mural thrombosis has not been identified previously in midventicular ballooning as opposed to apical ventricular ballooning syndrome [7].

Patients with dynamic intraventricular obstruction require beta blocker therapy and phenylephrine infusion can be considered in addition. Nondihydropyridine calcium channel blocker can be used to treat epicardial coronary spasm [2,3]. The outcome of patients with transient left ventricular dysfunction is generally good with 10% recurrence rate and 1% inpatient mortality rate. The recovery of the left ventricular systolic function is invariable after days to few weeks; however these patients may continue to have pathologic Q waves on the surface electrocardiogram. [1,2].

## Conclusion

Transient left ventricular ballooning dysfunction is an important differential diagnosis in patients presented with acute coronary syndrome (ACS) especially in older women. In the emergent setting it is treated as ACS, when it is suspected thrombolytic therapy should be withheld, primary coronary angioplasty becomes the preferred method for diagnosis and intervention when needed.

## Abbreviations

ACS: Acute Coronary Syndrome.

## Consent

Written informed consent was obtained from the patient for publication of this case report and accompanying images. A copy of the written consent is available for review by the Editor-in-Chief of this journal.

## Competing interests

The authors declare that they have no competing interests.

## Authors' contributions

JYA drafted the manuscript, CBH and SKH reviewed and proof read the manuscript, MS, RH, and CBH assisted in including references, locating and formatting images, and in final revisions of the manuscript.
